# Pericarditis Induced by Methotrexate: A Case Report and Literature Review

**DOI:** 10.7759/cureus.40257

**Published:** 2023-06-11

**Authors:** Nehemias A Guevara, Tabata E Hernandez, Flor Rosado, Sorab Gupta

**Affiliations:** 1 Internal Medicine, SBH (St. Barnabas Hospital) Health System, Bronx, USA; 2 Oncology, Albert Einstein College of Medicine, Bronx, USA

**Keywords:** pericarditis, systemic inflammatory disease, pericardial effusion, methotrexate, rheumatoid arthritis

## Abstract

Methotrexate (MTX) is a crucial part of the management of rheumatoid arthritis (RA). Although MTX is generally well tolerated, it is not exempt from adverse effects. The side effects of MTX are diverse, and pericarditis, as demonstrated in our case, is one of the rarest adverse effects. Therefore, it is crucial to rule out common causes of pericarditis, such as infections, malignancies, or other underlying conditions. However, once common causes are excluded, MTX-induced pericarditis should always be considered, and MTX should be discontinued without resumption due to potential relapse. Furthermore, the timing of MTX initiation is not significant; patients may have been on chronic MTX treatment or have recently started it. Moreover, pericarditis secondary to MTX is not exclusive to patients with rheumatological conditions but can also occur in other conditions.

## Introduction

Rheumatoid arthritis (RA) is a systemic inflammatory disease associated with high mortality and a relatively poor prognosis. Factors associated with a worse prognosis of the disease include positive anti-citrullinated antibodies with seropositive rheumatoid factor, anemia of chronic disease, and extra-articular manifestations [1]. Methotrexate (MTX) is a chemotherapeutic and immunosuppressive drug commonly used for RA treatment. However, it is associated with multiple side effects. Serositis is a rare complication of MTX therapy, with pleuritis reported earlier and more frequently than pericarditis.

The side effects spectrum of MTX is broad, but pericarditis has been reported only a few times, with the first case reported in 1995 in a pregnant woman treated for a molar pregnancy [2]. The most common side effects are mucositis and hematologic abnormalities. However, these effects are dose-dependent and mainly observed at high doses [3]. In this case report, we present a case of pericarditis and conduct a systematic literature review on pericarditis associated with MTX.

## Case presentation

A 55-year-old Hispanic male with a medical history of hypertension, type 2 diabetes mellitus, atrial fibrillation, and seropositive RA (on long-term MTX) presented to the emergency department with a complaint of chest discomfort for two weeks. The patient described the pain as sharp, located in the middle of his chest, with an intensity of 3-5/10, non-radiating, accompanied by shortness of breath, worsened by lying on his back, and relieved by sitting. The review of systems was negative, and he denied recent travel. His exercise tolerance was limited at baseline due to RA. He received adalimumab injections every two weeks and had been on a weekly dose of MTX (15 mg/weekly) for two years before this presentation. Upon arrival, his vital signs were unremarkable. During the physical examination, he appeared to be in mild distress due to chest discomfort, had tachycardia with jugular venous distention, bibasilar lung crackles, distant cardiac sounds, and no pericardial rub. The patient had grade III bilateral lower extremity swelling and mildly tender bilateral rheumatoid nodules scattered over the extensor surface of the proximal forearms with associated contractures of several digits. The physical exam was notable for mild tenderness and edema in several metacarpophalangeal (MCP) joints and ankles, along with mild swelling of multiple bilateral edema of proximal interphalangeal (PIJ) and distal interphalangeal joints (DIJ) and hammertoes deformity.

Findings and management

**Figure 1:**
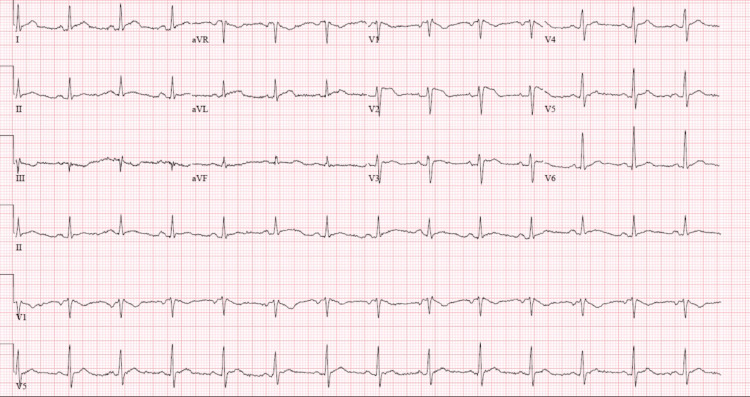
Electrocardiogram (EKG) showed nodal sinus rhythm (NSR) and possible left atrial enlargement

On arrival, an electrocardiogram (EKG) showed nodal sinus rhythm (NSR) and possible left atrial enlargement (Figure 1). A chest X-ray showed cardiac enlargement with a subtle left pleural effusion and subsegmental atelectasis. An echocardiogram (IFigures 2-3) revealed moderate to large pericardial effusion with early signs of tamponade and an ejection fraction of 65%. Computed tomography angiography (CTA) demonstrated a large pericardial effusion measuring up to 2.5 cm thick with cardiomegaly and mild coronary arteries calcifications, subsegmental atelectasis, mild bilateral pleural effusion, and multiple small mediastinal nodes, with the rest unremarkable (Figures 4). Troponins were negative, Complete blood count (CBC) was unremarkable, and brain natriuretic peptide was elevated at 54 pg/mL (Table 1).

**Figure 2:**
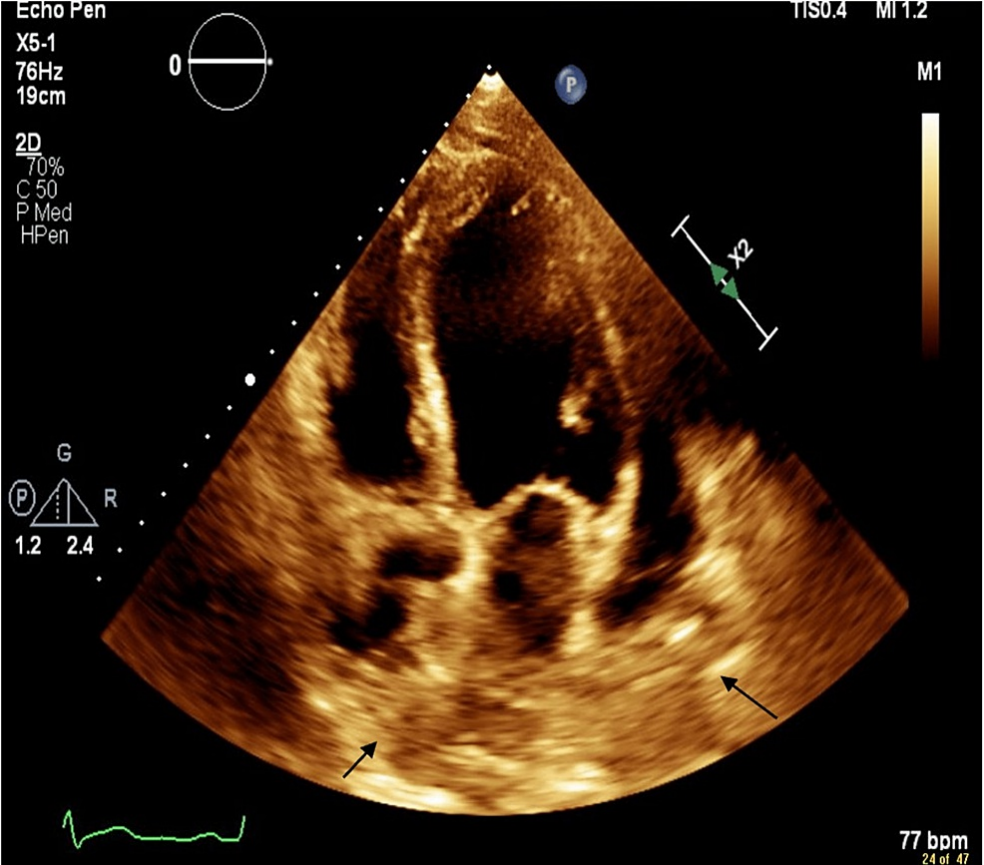
Echocardiogram apical four-chamber view (black arrows are pointing to the pericardial effusion)

**Figure 3:**
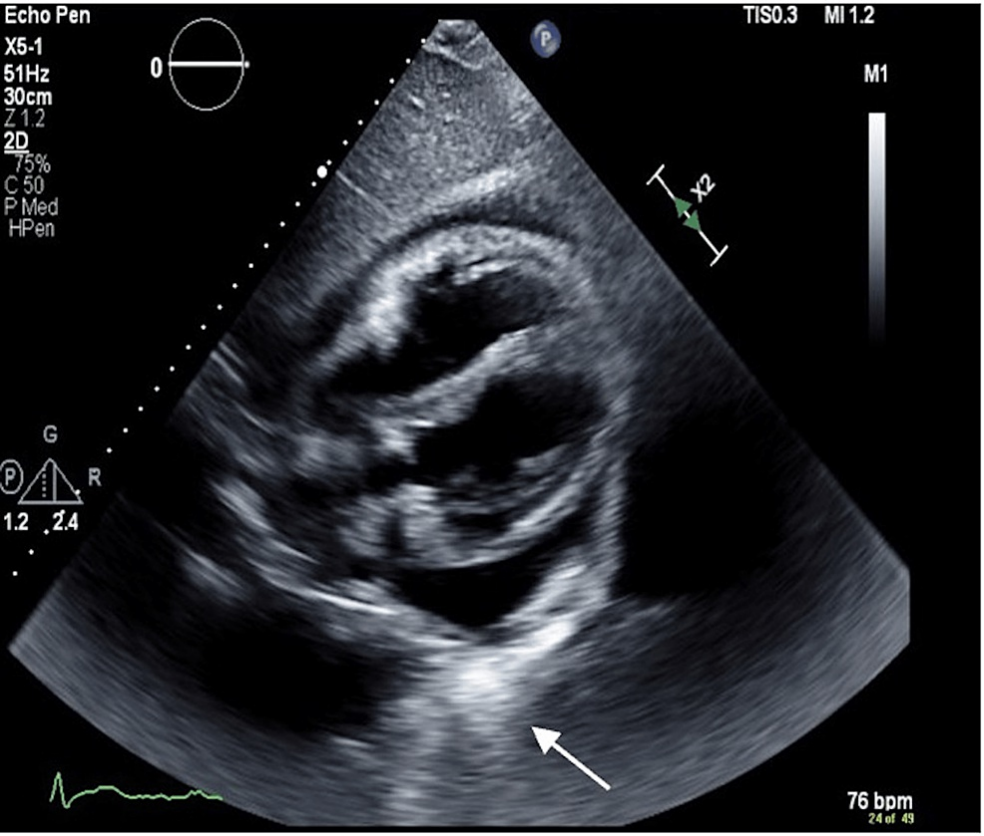
Echocardiogram subcostal four-chamber view (white arrow is pointing to pericardial effusion)

**Figure 4:**
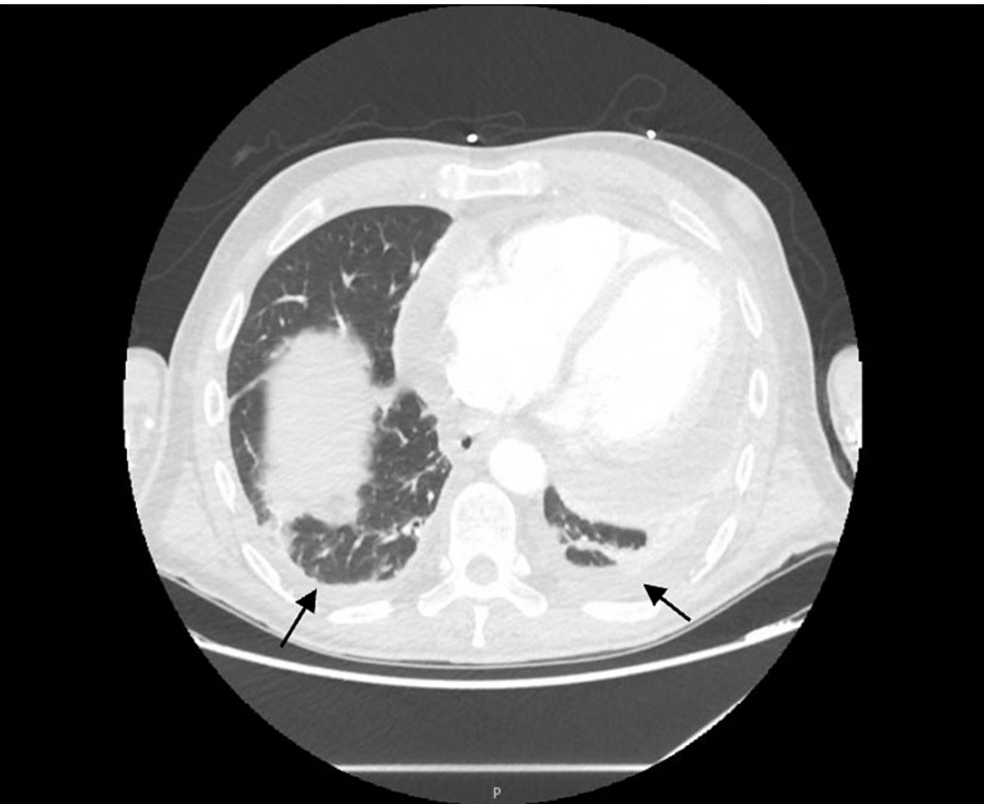
CTA chest (black arrows are pointing to large pericardial effusion) CTA: computed tomography angiography

**Table 1:** Laboratory values P-ANCA: perinuclear anti-neutrophil cytoplasmic antibodies; C-ANCA: antineutrophil cytoplasmic autoantibody, cytoplasmic; dsDNA: double-stranded DNA *QIAGEN, Hilden, Germany

Laboratory	Value	Reference value
Brain natriuretic peptide (BNP)	65	0-100 pg/mL
Erythrocyte sedimentation rate (ESR)	>100	0-14 mm/hr
Rheumatoid factor (RF)	Positive	Negative
Cyclic citrullinated peptide (CCP) antibody IgG/IgA	>250	0-19 Units
Anti Myeloperoxidase (MPO) antibodies	<9	0.0-9.0 U/mL
Antiproteinase 3 (PR-3) antibodies	<3.5	0-3.5 U/mL
Complement, C3/ C4	71/7	82-167 / 14-44 mg/dL
Anti dsDNA	Negative	Negative
QuantiFERON-TB Gold*	Negative	Negative
Antineutrophil cytoplasmic antibody P-ANCA/C-ANCA	<1:20	Neg <1:20
Hepatitis C	Negative	Negative/Positive
Hepatitis B	Positive surface and Core antibody, negative surface antigen	Negative/ Positive

**Figure 5:**
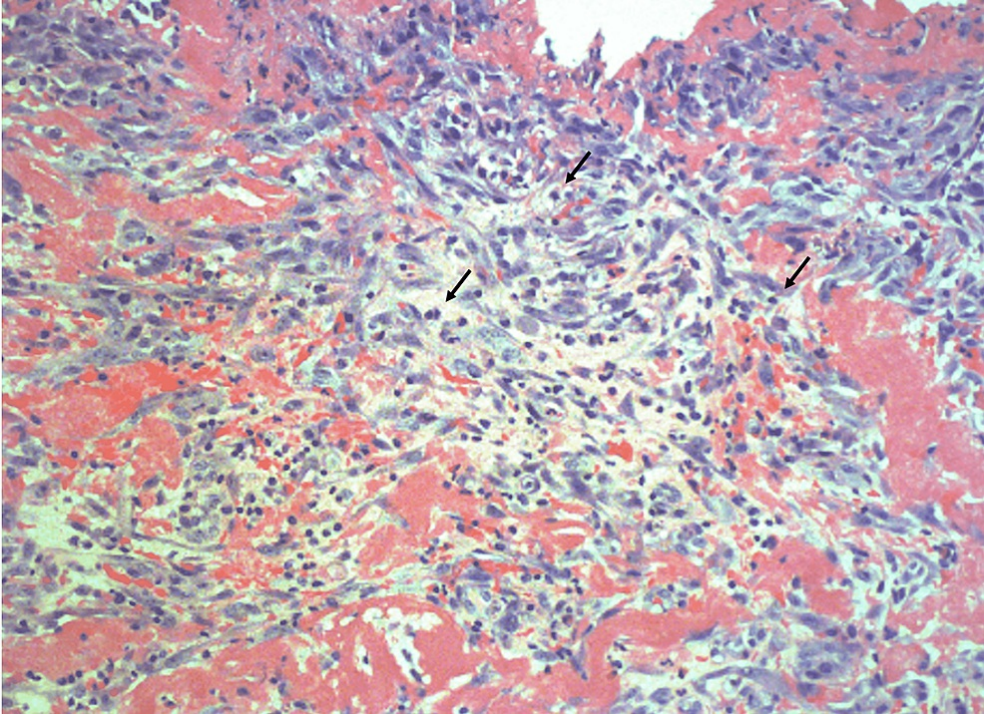
Pericardial biopsy revealed chronic inflammation (black arrow pointing to lymphocytes) and fibrin infiltration of inflammatory cells. Additionally, there were signs of fibrosis; no evidence of malignant cells was found.

On admission, adalimumab and MTX were discontinued due to a possible association with pericarditis. However, adalimumab was later restarted in the outpatient setting, but MTX was not resumed. Due to the concerns of early signs of tamponade, the patient underwent a left anterior thoracotomy performed by cardiothoracic surgery. During the procedure, 300 cc of serosanguineous fluid was drained, and a pericardial biopsy was performed. A pericardial window was created and left in place for three days, allowing for the drainage of approximately 300 cc of fluid with a similar appearance. The cytology report was negative for malignant cells but showed numerous red blood cells. Pathology results indicated fibrinous pericarditis (Figure 5), with findings of fibrin, fibrosis, and chronic inflammatory cell infiltrate, including small lymphocytes and eosinophils, without granuloma or malignancy. The patient has been following up with rheumatology in the outpatient setting, and serositis has not recurred after the discontinuation of MTX.

## Discussion

MTX belongs to the group of folate antagonists and was initially developed as an antineoplastic agent for the treatment of certain cancers, particularly hematologic malignancies. It is also a disease-modifying agent used to treat various autoimmune pathologies, including RA. MTX possesses anti-inflammatory and immunomodulatory properties and is one of the essential components in managing RA [4,5].

MTX exerts its effects through diverse mechanisms. One of the most well-known mechanisms is the inhibition of the enzyme dihydrofolate reductase, which, at high doses (1 gram in a single dose), leads to the inhibition of DNA synthesis and cell cycle arrest in the S phase, ultimately resulting in cell apoptosis [6,4,7]. Additionally, it affects the adenosine deaminase signaling pathway, particularly when used at lower doses (ranging from 15 mg to 25 mg weekly) for the treatment of RA and other autoimmune conditions [4,8]. In this pathway, MTX blocks the enzyme 5-aminoimidazole-4-carboxamide ribonucleotide transformylase (ATIC), which is responsible for the conversion of imidazole carboxamide ribonucleotide (AICAR) to 5-Formamido Imidazole-4-carboxamide riboside. By inhibiting this enzyme, MTX increases AICAR levels within the cell, inhibits adenosine deaminase activity, decreases the breakdown of adenine to inosine, and enhances extracellular adenosine levels, promoting the activation of signaling pathways. Patients with RA often exhibit overexpression of adenosine deaminase receptors in immune cells, particularly monocytes, and macrophages, which play a crucial role in regulating and inhibiting the release of pro-inflammatory cytokines [3,9,10].

The dosage of MTX can influence the spectrum of adverse effects. Factors such as gastrointestinal absorption, excretion rate, co-administration with other drugs, and inter-individual variability can impact both the efficacy and side effects of MTX. High doses of MTX can precipitate renal, neurological, hematologic, pulmonary, and gastrointestinal toxicities, potentially leading to life-threatening conditions. Leucovorin can be used as a rescue therapy where high doses of MTX are administered [10]. In contrast, lower doses of MTX are associated with hepatic fibrosis, fatty liver, hepatic cirrhosis, gastrointestinal manifestations, mucocutaneous lesions, and pulmonary fibrosis [11,12].

As a cornerstone in the treatment of RA, MTX is typically administered for an extended duration. The side effect profile may vary depending on the concentration and frequency of drug administration. Several studies have shown that higher initial doses of 25 mg/week are associated with a higher incidence of gastrointestinal manifestations compared to lower doses of 15 mg/week [13]. The most commonly described side effects include elevation of liver enzymes, cytopenia, opportunistic infections, dermatologic manifestations, dry cough, interstitial pneumonitis, memory impairment, nausea, headache, dizziness, and serositis [14,15]. However, pericarditis associated with MTX use is extremely rare.

The present case report describes a patient who developed pericarditis as an isolated phenomenon following long-term treatment with MTX. The patient had received a stable low dose of MTX for over two years before presenting to the hospital with pleurisy, shortness of breath, and chest pain that worsened with leaning forward. Imaging revealed moderate pericardial effusion, confirmed by echocardiography and accompanied by early signs of cardiac tamponade. Subsequently, the patient underwent pericardial drainage via a pericardial window procedure.

Notably, the patient developed moderate pleural effusion, more frequently associated with pericarditis, despite being managed with MTX for an extended period without significant drug regimens or dose changes. The pericardial fluid analysis was negative for infectious or malignant causes. Histopathological examination of the pericardium showed fibrin, fibrosis, chronic inflammatory cell infiltrates, small lymphocytes, and eosinophils, consistent with fibrinous pericarditis. MTX was immediately discontinued, and the patient was closely monitored for two years with no recurrence of pericardial fluid or associated symptoms. These findings suggest that MTX most likely induced the patient's pericarditis. After discharge, the patient was re-started on adalimumab with the same dose, but MTX was not reintroduced, and no recurrence of pericarditis was observed. These findings suggest that MTX most likely induced the patient's pericarditis.

In our research, we found a compilation of eight case reports focusing on pericardial effusion associated with the use of MTX within the last 15 years. In three of the cases, pericarditis resulted from using MTX alone, and in five cases, there was reported the use of an additional drug along with MTX; these medications included topical steroids, amoxicillin, ceftriaxone, azithromycin, hydrochlorothiazide celecoxib, amlodipine, esomeprazole, methylprednisolone, acetylsalicylic acid, colchicine, daunorubicin, vincristine, prednisone, dexamethasone; asparaginase was reported to be used at the same time. Any of these drugs have previously been described to be associated with pericarditis as part of their side effect profile. The ages varied from 7-62 years; the average age was 42 years old. Three out of eight cases described above were female, and five out of eight. cases were male. The primary diagnoses associated with these cases were rheumatoid arthritis [1,16], molar pregnancies [2,17], psoriatic arthritis [13,18], cutaneous psoriasis [19], and T-cell lymphoma [20]. The diagnosis of pericardial effusion was typically made using clinical and imaging techniques, such as echocardiography, chest X-ray, computerized thoracic tomography, and electrocardiography. Clinical findings were often the initial method used to raise a suspicion of pericardial effusion, with less common diagnostic tools, including bronchoscopy and a high-resolution CT scan of the chest. Among all the cases, the most common comorbidity was hypertension, which was reported in three out of eight patients; other less frequent comorbidities were gastrointestinal reflux disease, polycystic kidney disease, and sinusitis; and four out of eight did not report any comorbidity. Additional complications associated with the administration of MTX, nine patients exhibited concurrent pleural effusion alongside pericardial effusion. Less common complications included hepatotoxicity, dense adhesions resulting in thickened pleura, cardiomegaly, and atelectasis. Various interventions were implemented, such as pericardiocentesis, aspiration of pericardial or pleural fluids, discontinuation of MTX, and the utilization of oral steroids, antimicrobial therapy, or a combination of both. The details of the reviewed cases are given in Table 2.

**Table 2:** Summary of reviewed cases

References	Associate drugs	Patient Age	Patient Gender	Main Disease	Comorbidities	Associate complications	Diagnostic tools	Interventions and treatment	Cytology	Time of resolutions	Recurrences after d/c	Time of Methotrexate use
Palungwachira et al., 1998 [13]	Topical crude coal tar and topical corticosteroid	62 years old	Female	Psoriasis	None reported	Pericarditis and Pericardial effusion	Not reported	Aspiration of pericardial fluid	Not reported	Not reported	None after six months of follow-up	Not reported
Forbat et al., 1995 [2]	None	22 years old	Female	Molar pregnancy	None	Pleurisy, Pneumonitis, Pericardial effusion	Clinical, EKG, Echo, Chest x-ray	Adequate hydration and naproxen pericardiocentesis	650 clear fluid abundant lymphocytes, reactive mesothelial cells, and scattered eosinophils, but no malignant cells	Not reported	None after 18 months of follow-up	16 weeks
Dündar et al., 2013 [17]	Folinic acid in between each methotrexate dose Antibiotics	33 years old	Female	Hydatidiform mole	Sinusitis	Hepatotoxicity, Pericardial effusion	Clinical, EKG, Echo, Chest CT scan	Pericardiocentesis	Microbiologically, cytologically and biochemically, which did not reveal any abnormalities .	Three months after pericardiocentesis. Methotrexate was not discontinued second course was delayed 14 days	None after one-year follow-up	Four doses in one week
Cudzilo et al., 2014 [18]	Oral Prednisone	41 years old	Male	Psoriatic arthritis	Polycystic kidney disease, Hypertension	First episode of pleural effusion (Left); Second episode of pleural effusion (Right); Dense adhesions and thickened pleura. Pericardial effusion	Clinical, Chest x-ray, Echo	Thoracentesis Pericardiocentesis	Exudate with 30% eosinophils (1st thoracentesis) Hemorrhagic fluid 79% neutrophils in pericardial fluid Exudate with 5% eosinophils (2nd thoracentesis)	Two weeks after discontinuation	Not reported	One month and then discontinued for unclear reasons; Restarted two episodes after eight weeks of methotrexate use
Whitfield et al., 2020 [1]	Oral Prednisone	52 years old	Male	Rheumatoid arthritis	Hypertension	Large right pleural effusion Pericardial effusion	Clinical, Chest x-ray, Echo	Methylprednisolone 125 mg x 5 days, Ceftriaxone 2 grams x 10 days, Azithromycin 500mg x 7 days, Thoracentesis with subsequent placement of two chest tubes Pericardiocentesis	Not reported	10 days after decreased dose of Methotrexate posteriorly discontinued and switched to Abatacept	No reported	(9) 3 years
Savoia et al., 2010 [19]	No reported	57 years old	Male	Cutaneous psoriasis	None	Aseptic pleuro-pericarditis	Clinical, Chest x-ray, Echo, EKG, Bronchoscopy, Extensive blood work for infectious causes, Chest high-resolution CT scan	Thoracentesis Acetylsalicylic acid 500 mg daily Colchicine 1 mg daily	Negative for bacteria, mycobacteria, and neoplastic cells	20 days after discontinuation	None after 10 months of follow-up	1 month
Thomas et al [20]	Daunorubicin Vincristine Prednisone Dexamethasone Asparaginase	7 years old	Male	T-cell lymphoblastic lymphoma	None	Mild cardiomegaly Pericardial effusion	Clinical, Chest x-ray, Echo	Anti-inflammatories for 1st pericardial effusion with resolution	Protein 6.27g/dl, Adenosine deaminase, and glucose 107.6 mg/dl, the fluid paucicellular with inflammatory cells	Not reported	Not reported	1 day
Mohududdin et al [16]	Hydrochlorothiazide, Amlodipine, Celecoxib, Esomeprazole.	60 years old	Male	Rheumatoid arthritis	Hypertension Gastro-intestinal reflux disease	Atelectasis Pericardial effusion	Clinical, EKG, Echo	Prednisone 30mg daily	No reported	Not reported	None after three years of follow-up	1.5 years

The cytology of the pericardial or pleural fluids revealed various findings, including reactive mesothelial cells, scattered eosinophils, lymphocytes, and mild chronic inflammation. Among the cases that reported the amount of pericardial fluid drained, the average volume was 1,016 ml, with a minimum of 400 ml and a maximum of 2,000 ml. In six out of eight cases, the cytology of the pericardial fluid described serous fluid with white blood cell count within the normal range, normal glucose level, and no evidence of malignancy or infection. Only one case reported a biopsy showing prominent eosinophils and mild chronic inflammation [2]. It is important to note that all cases had negative cytologic reports for malignancy and that both infectious and inflammatory causes were excluded in every case.

The average time for symptom resolution range between two to three weeks following the discontinuation of MTX. Remarkably, some patients remained symptom-free even during long-term follow-ups, which ranged from 1-18 months. In one case, steroids were required in addition to the discontinuation of MTX. Only one patient experienced pericardial and pleural effusion recurrences, while MTX was not discontinued, but symptoms resolved after discontinuation. It is important to note that, while these cases suggest an association between the use of MTX and pericardial or pleural effusion, further research is needed to establish a causal relationship. Close monitoring of patients using MTX for potential adverse events such as pericardial effusion is crucial, and any symptoms should be reported to their healthcare provider [1,13,16-20].

## Conclusions

Pericarditis can occur as a rare side effect of MTX in patients with rheumatological conditions and other medical conditions. MTX is associated with a wide range of side effects; although rare, pericarditis has been observed in our case. Therefore, it is essential to thoroughly evaluate and rule out more common causes of pericarditis, such as infections, malignancies, or the underlying disease itself. However, once these common causes have been excluded, MTX-induced pericarditis should always be considered, leading to the discontinuation of the medication. Furthermore, the timing of MTX initiation is not significant, as patients may have been on chronic treatment or recently started. It is advised to avoid resuming MTX due to the potential risk of recurrence.
